# Validity Evidence for the Better Research Interactions for Every Family (BRIEF) Assessment Tool

**DOI:** 10.7759/cureus.113658

**Published:** 2026-07-30

**Authors:** Michelle J Bartlett, Stephanie A Kraft, Megan M Gray, Devan Duenas, Sandra E Juul, Elliott M Weiss

**Affiliations:** 1 Division of Neonatology, Department of Pediatrics, Children's Hospital of Philadelphia, Philadelphia, USA; 2 Department of Bioethics and Decision Sciences, Geisinger College of Health Sciences, Danville, USA; 3 Division of Neonatology, Department of Pediatrics, University of Washington School of Medicine, Seattle, USA; 4 Treuman Katz Center for Pediatric Bioethics and Palliative Care, Seattle Children's Research Institute, Seattle, USA

**Keywords:** assessment tool validity evidence, informed consent education, messick validity framework, neonatology clinical trial recruitment, neonatology clinical trials, patient researcher communication

## Abstract

Background

Recruiting families for neonatal clinical trials is challenging due to narrow enrollment windows, high clinical acuity, and parental stress, which contributes to concerns regarding representativeness in neonatology research. The Better Research Interactions for Every Family (BRIEF) intervention was developed to improve recruitment experiences and equity. We aimed to gather validity evidence using Messick’s framework for the BRIEF assessment tool, a component of the BRIEF educational intervention, designed to evaluate research team members’ consent and recruitment interactions.

Methods

In this pilot prospective cohort study, physicians participating in the BRIEF educational intervention during a neonatal clinical trial completed recorded consent discussions (n = 22). Two blinded expert raters then evaluated sessions using the 10-item BRIEF assessment tool, a component of the BRIEF educational intervention. Participants completed self-assessments and surveys. Validity evidence for the BRIEF assessment tool was evaluated using weighted kappa for inter-rater agreement, Wilcoxon rank-sum tests for associations with self-reported skill, and Cronbach’s alpha for internal consistency.

Results

Five physicians contributed 22 recorded encounters using the BRIEF assessment tool. Internal consistency was strong across items (α > 0.70). Inter-rater agreement ranged from poor to almost perfect, with half of the items demonstrating moderate or higher agreement. Agreement between self-assessment and expert ratings was poor. Only one performance objective (PO) correlated with self-reported skill. Lower expert-rated scores were associated with families declining trial participation. Most participants rated themselves higher than expert raters.

Conclusions

In this pilot study, the BRIEF assessment tool demonstrates preliminary validity as an observer-based measure of neonatal research consent interactions, with strong internal consistency and variable inter-rater reliability. Self-assessment utility appears limited. Further validation in larger, more diverse samples is needed before high-stakes use.

## Introduction

Recruiting participants for clinical trials can be challenging across all of medicine; however, neonatology has unique considerations for discussing research with families expecting or with newborns [1]. Consent conversations are often complicated by narrow enrollment windows and high clinical acuity, as well as parental emotional and physical stress [2,3]. Infants enrolled in neonatology research studies may be sicker than infants who are not enrolled and therefore not representative of the general population [4]. Together, these factors contribute to ongoing difficulties with representativeness within neonatal clinical trials [5].

Approaching families about neonatal clinical trials and facilitating informed consent discussions are difficult tasks. Research team members must be prepared to respond to research-related concerns as well as clinical and emotional factors relevant to the experience of eligible families [6]. In order to address the need for improved recruitment processes, the Better Research Interactions for Every Family (BRIEF) educational intervention was developed and has been previously published [7]. The overall aims of the BRIEF educational intervention were to improve the recruitment experience for families as well as increase enrollment in clinical trials in neonatology, with a particular focus on the needs of families from minoritized backgrounds and reducing disparities in neonatology research participation [7]. Parent experience and research team member feedback from the single-centered pilot of BRIEF have been previously reported [8,9].

As new educational interventions are created, it is paramount to collect ongoing evaluation data of the intervention. As a component of the BRIEF educational intervention, the BRIEF assessment tool was created to collect this evaluation data. The purpose of the BRIEF assessment tool was to evaluate research team members in their task of recruitment and consent conversations with families. In order to provide rigorous feedback for the intervention and the research team participants, a tool should be able to reliably evaluate the quality of the conversations between families and research teams. The objective of this study was to evaluate the validity of the newly developed BRIEF assessment tool using Messick’s validity framework [10,11].

## Materials and methods

BRIEF intervention

The development of the BRIEF educational intervention, including curricular elements and design, has been previously published (Appendix) [7]. The BRIEF assessment tool was developed as part of the BRIEF educational intervention (Appendix) [7]. The BRIEF intervention was developed using intervention mapping, which focuses on theory-informed intervention development, implementation, and evaluation [7,12]. There was extensive input from existing literature, content experts, and families. The BRIEF intervention focused on research team members who perform recruitment and consent conversations and included online prework as well as an in-person group session including role-play [7]. Further curricular details can be found in the previously published work [7]. The intervention had 10 performance objectives (POs) that were a part of a larger four-stage framework: (1) pre-approach, (2) initial connection, (3) building connection, and (4) follow-up (Table 1) [7]. The intervention was piloted as an ancillary study to the darbepoetin plus slow-release intravenous iron (DIVI) neonatal clinical trial [13]. The overall evaluation plan for the BRIEF intervention included multiple levels of outcomes; the BRIEF assessment tool was one level of outcome specifically designed to evaluate the interaction and consent conversation between parents and research team members [7].

**Table 1:** Conceptual framework and related POs for the BRIEF assessment tool PO: performance objective; BRIEF: Better Research Interactions for Every Family

Stage	PO
Pre-approach	PO.1 Partnership with clinical team
Initial connection	PO.2 Partnership with bedside nursing
	PO.3 Family names
	PO.4 Options for discussing research
	PO.5 Empathy with the NICU family experience
	PO.6 Family needs
Building connection	PO.7 Research team’s investment in trial
	PO.8 Benefit for future infants
	PO.9 Options for participation
Follow-up	PO.10 Ongoing connection with family

BRIEF Assessment Tool Development

The development of the BRIEF assessment tool started with a literature review of published best practices related to informed consent and an open discussion among our author group of experts in neonatology, bioethics, and clinical research based on the initial framework and POs as described and published [7]. A global rating scale (GRS) format was chosen that provides examples of skills along a continuum rather than a yes/no checklist format to better capture the complexities and nuances within a neonatal clinical research environment [14,15]. The BRIEF assessment tool included 10 POs; each linked to a specific behavior (Appendix). The BRIEF assessment tool used a Likert scale from 1 to 5, with 1 being more novice and 5 being more expert (Appendix). Expert raters were instructed to select which level (including half-levels) best matched their perception of the recruitment session.

Study design

This study utilized a pilot prospective cohort of physicians who performed informed consent conversations, evaluated using the BRIEF assessment tool, as a part of the BRIEF educational intervention during DIVI trial recruitment at the University of Washington School of Medicine. Participation was voluntary. Recordings were done on a study iPad after the consenter obtained permission from the prospective family participant. Only the members of the research team who had consent conversations during the specific time period of BRIEF assessment tool implementation were included. Identifying information (such as names) was removed from recordings; however, voices were unable to be masked. Two independent expert raters evaluated each recording, blinded to the self-assessment scores. Raters were experts in pediatric research ethics, informed consent, and neonatology who previously co-developed the tool and jointly agreed upon guidance for connecting the label to a typical learner. The tool also provides guidance and examples for the raters to use to guide their scoring (Appendix). Participants completed a survey that included demographic information, experience with performing informed consent, and feedback related to the BRIEF assessment tool. This was a pilot study, so no sample size was calculated. This study was approved by the University of Washington IRB.

Data analysis

Rater and participant survey responses were collected and stored by the REDCap electronic data capture tool for numerical data hosted at the University of Washington. Participant surveys and expert rater assessment scores on the BRIEF assessment tool were analyzed using descriptive statistics. A weighted kappa test was utilized for inter-rater agreement between expert raters as well as between expert raters and participant self-assessments; kappa outputs were classified as poor (0), slight (0.01-0.20), fair (0.21-0.40), moderate (0.41-0.60), substantial (0.61-0.80), almost perfect (0.81-0.99), and perfect (1.0). A Wilcoxon rank sum test (p < 0.05) was performed to evaluate for correlation between expert rater 1’s PO score and participant self-identified skill performing these conversations to evaluate whether the ratings scale with a relevant skill. Intraclass correlation (ICC) with Cronbach’s alpha (α > 0.70) was used to evaluate inter-item correlation. Inter-rater reliability across the 10 items was evaluated using a cross-classified linear mixed-effects model via restricted maximum likelihood (REML) estimation. This multilevel approach was selected to simultaneously control for the crossed random effects of subjects (IDs), questions, and raters, avoiding data inflation or loss of granularity caused by traditional aggregation. The inter-rater intraclass correlation coefficient was calculated by isolating the subject-level variance component against the total variance. Due to a high concentration of scores within specific categories on the rating scale, Cohen’s Kappa suffered from the well-documented prevalence paradox, penalizing the reliability index despite a moderate percent agreement for the comparison of expert vs self-ratings. Therefore, Gwet's AC2 with linear weights was utilized as a paradox-resistant alternative to provide a more stable and accurate estimation of true inter-rater agreement for that comparison. Statistical analysis was done with Stata software release 17 (Stata Corp 2021; StataCorp LLC, College Station, TX).

## Results

Of the total of nine DIVI team members who participated in the BRIEF educational intervention, five physicians had recruitment and consent discussions with families audio-recorded for a total of 22 informed consent conversations evaluated using the BRIEF assessment tool (10 pre-BRIEF, 12 post-BRIEF). These participants had between one and 11 recorded consent conversations, each with a median of 2. The demographic information of the participants is listed in Table 2, and the demographic information of the families of the infants approached for consent is listed in Table 3. Due to strict eligibility criteria for DIVI, some infants enrolled prenatally were not eligible for the study at the time of birth.

**Table 2:** Demographics of physicians (N = 5) with audio-recorded DIVI consent discussions DIVI: darbepoetin plus slow-release intravenous iron; IQR: interquartile range

	N (%)
Years in current role (median with IQR)	4 (2.2-18.5)
Years in clinical research (median with IQR)	8.5 (6-17)
Number of clinical research trials consented for in past (median with IQR)	3.5 (1.5-4.75)
Gender	
Female	4 (80%)
Male	1 (20%)
Age in years (median with IQR)	40.5 (39-60)
Race/ethnicity	
White	5 (100%)
Research recruitment experience	
Some amount of experience	3 (60%)
Moderate amount of experience	1 (20%)
Frequently supervise others in recruitment	1 (20%)
Comfort with approaching potential participants for clinical research	
Extremely comfortable	1 (20%)
Moderately comfortable	4 (80%)
Self-reported skill level in approaching potential participants for clinical research	
Moderately to very skilled	3 (60%)
Slightly skilled	2 (40%)
Previous training in consent conversations for clinical research	
On the job learning	5 (100%)
Didactic training for a specific study	1 (20%)
Being observed by others and getting feedback	1 (20%)
Observing experienced team members	3 (60%)
Satisfaction with prior training in clinical research recruitment	
Moderately satisfied	3 (60%)
Slightly satisfied	2 (40%)

**Table 3:** Demographics of infant and families (N = 22) with audio-recorded DIVI consent discussions DIVI: darbepoetin plus slow-release intravenous iron; IQR: interquartile range; PPV: positive pressure ventilation; CPR: cardiopulmonary resuscitation

	N (%)
Consent conversation timing	
Prenatally	3 (14%)
Postnatally	19 (86%)
Plurality (number of fetuses in single pregnancy)	
Singleton	16 (73%)
Multiple	6 (27%)
Medicaid status within the past year	
Yes	18 (82%)
No	3 (14%)
Unable to determine	1 (4%)
Race	
American Indian or Alaska Native	2 (9%)
Asian	2 (9%)
Black	3 (14%)
White	13 (59%)
Declined to provide race	2 (9%)
Ethnicity	
Hispanic/Latino/Spanish	2 (9%)
Declined to provide ethnicity	2 (9%)
Laboring parent complications & treatments	
Chorioamnionitis	3 (14%)
Antibiotics	9 (41%)
Magnesium sulfate	19 (86%)
Prenatal steroids	20 (91%)
Rupture of membranes >18 hours	6 (28%)
Delivery mode	
Vaginal	2 (9%)
Cesarean	20 (91%)
Birth weight in grams (median with IQR)	1277 (970-1685)
Completed weeks of gestation (median with IQR)	30 (27-31)
Birth location	
Inborn	21 (95%)
Outborn	1 (4.5%)
Infant sex	
Female	8 (36%)
Male	14 (64%)
Delivery room treatments	
PPV	15 (68%)
Intubation	7 (32%)
CPR	0 (0%)
Enrollment and participation	
Enrolled and participated	14 (64%)
Declined enrollment	4 (18%)
Declined participation after enrollment	1 (4.5%)
Became in-eligible before participation	3 (13%)

Content validity

Within Messick’s framework, content validity describes how well the elements of an assessment tool represent the underlying construct the tool is designed to measure [11]. The BRIEF assessment tool was developed as a part of a rigorously prepared educational intervention that has been previously published [7]. Content was developed based on a thorough literature review, expert consensus, and parental input using an intervention mapping strategy and social cognitive theory (SCT) [7]. SCT posits that behavior change occurs through the alignment of outcome expectations, beliefs about the results of an action, and self-efficacy expectations, or confidence in one’s ability to perform that action [16]. In the BRIEF intervention, four SCT determinants were used: behavior capability, self-efficacy, outcome expectations, and subjective norms [7]. A conceptual framework, based on a prior empirical analysis, was used that included four stages: pre-approach, initial connection, building connection, and follow-up [17]. The content expert panel and parent advisory panel then met multiple times and iteratively revised a list of 16 initial items to a final set of 10 POs, which were then linked to SCT determinants (Table 1, Appendix) [7].

Relationship to Other Variables

Validity evidence for relationships to other variables examines whether scores on the assessment tool correlate with the underlying construct being measured; this concept aligns with the construct validity framework described by Cronbach and Meehl [11,18]. When comparing participant self-reported skill level to expert rater PO assessment scores on the BRIEF assessment tool, there was only one statistically significant value: PO.3 Family names (Table 4). This indicates that participant self-reported skill at approaching families for consent in clinical trials did not correlate with expert rater-scored assessments on the tool. Parent experiences and enrollment outcomes have been previously published; most parents reported a positive experience with the POs being measured by the BRIEF assessment tool; however, the study was not powered to identify differences in enrollment or parental experience after the BRIEF intervention was implemented [8]. Lower expert rater PO scores on the tool were associated with parents declining participation in the DIVI trial, which suggests that these POs may be relevant to parental decision to participate in clinical trial research [8].

**Table 4:** Validity evidence: correlation between ratings and self-rated consenting skill, self and expert ratings, and between multiple expert raters PO: performance objective; κ: weighted kappa; NICU: neonatal intensive care unit Each assessment encounter (N = 22) was observed and scored by the same two expert raters. The p-value reflects expert rater score compared to participant self-identified skill level (Table 2) using a Wilcoxon rank sum test with p-values; p < 0.05 was considered statistically significant. n = 9 for recording of lower-rated skill participants and n = 13 for recording of higher-rated skills participants. Inter-rater reliability (agreement between expert raters) and criterion agreement (self-assessment versus expert rater) are reported as weighted κ and Gwets AC2, respectively, with strength of agreement interpreted per Landis and Koch: <0.00, poor; 0.01-0.20, slight; 0.21-0.40, fair; 0.41-0.60, moderate; 0.61-0.80, substantial; 0.81-1.00, almost perfect [19]

PO	Comparison of expert rater 1’s score with self-rated consenting skill level, Wilcoxon rank sum p-value (W statistic)	Level of rater agreement between expert raters 1 and 2 (weighted K coefficient, 95% CI)	Level of agreement between self-assessment and expert rater 1 (Gwets AC2 coefficient, 95% CI)	
PO.1 Partnership with clinical team	0.12 (85)	Almost perfect (0.85, 0.41-0.96)	Poor (-0.22, -0.53-0.08)	
PO.2 Partnership with bedside nursing	0.84 (105)	Fair (0.35, -0.25-1.00)	Moderate (0.51, 0.19-0.82)	
PO.3 Family names	0.04 (74)	Moderate (0.48, 0.29-0.59)	Fair (0.21, -0.10-0.53)	
PO.4 Options for discussing research	0.07 (86)	Moderate (0.43, 0.00-0.62)	Poor (-0.07, -0.34-0.20	
PO.5 Empathy with the NICU family experience	0.17 (84)	Moderate (0.43, 0.22-0.60)	Slight (0.17, -0.05-0.39	
PO.6 Family needs	0.88 (102)	Moderate (0.51, 0.31-0.58)	Fair (0.37, 0.06-0.69)	
PO.7 Research team's investment in trial	0.50 (113)	Fair (0.34, 0.22-0.48)	Fair (0.26, 0.07-0.45)	
PO.8 Benefit for future infants	0.41 (99)	Poor (0, 0.00-0.00)	Fair (0.38, 0.14-0.62)	
PO.9 Options for participation	0.11 (80)	Fair (0.39, 0.07-0.56)	Poor (-0.03, -0.44-0.37)	
PO.10 Ongoing connection with family	0.21 (86)	Fair (0.27, -0.02-0.45)	Slight (0.21, -0.13-0.55)	

Internal Structure

Internal structure is best defined as the consistency and relationship among data items, which is similar to the framework for reliability [11,19]. The cross-classified mixed model revealed poor overall inter-rater reliability, with an ICC of 0.20. The vast majority of the variance in the scores was driven by residual error (𝜎2 = 1.24), accounting for 64.8% of the total variance, indicating high random disagreement between the raters on an item-by-item basis. Systematic differences between the two raters themselves were negligible (𝜎2 < 0.001). Differences in baseline question difficulty accounted for 16% of the variance (𝜎2 = 0.30), while true differences between subjects explained only 19.5% of the variance (𝜎2 = 0.374). To complement the cross-classified mixed model, a standardized Cronbach's alpha was computed to assess the overall consistency between the two raters across all item responses. The analysis yielded a scale reliability coefficient of 𝛼 = 0.80, with an average inter-item correlation of r = 0.67, indicating a strong and cohesive linear relationship between the raters' scoring trends. Of the weighted kappas evaluating inter-rater reliability between expert raters for the 10 items, one had poor agreement (0), four had fair agreement (0.21-0.40), four had moderate agreement (0.41-0.60), and one had almost perfect agreement (0.81-1) (Table 4). 

Response Process

Response process refers to whether participants’ understanding and performance align with the construct as defined by the investigators [11]. During development of the BRIEF intervention, parental interviews were performed to ensure questions were interpreted as intended by participants [7]. Participant self-assessment as compared to expert rater assessment overall had poor reliability, with all elements being fair agreement or less (Table 4). Out of the total 220 measurements, 117 (53 %) of participant self- and expert rater assessments were within 1 point. A total of 85 (39%) participants rated themselves higher than the expert raters by more than one point and 18 (8%) below by more than one point.

Consequences

Consequences refer to the intended and unintended effects of using an assessment [11,20]. The BRIEF assessment tool did not have a pass or fail designation. Research team participant feedback on the BRIEF intervention has been previously published, and overall, the BRIEF intervention was found to be helpful [9]. Parents overall reported positive interactions with researchers [8].

## Discussion

Using Messick’s framework for validity (content validity, relationship to other variables, internal structure, response process, and consequences), we have presented validity evidence in a pilot study for the novel BREIF assessment tool, designed to assess neonatology research teams' clinical trial consent conversations with families [10].

The BRIEF assessment tool, within the larger BRIEF educational intervention, was developed through a rigorous intervention mapping process, lending content validity [7]. Expert rater assessment scores did not correlate with participant self-assessment scores or participant self-reported skill level, and when participant self-assessment PO scores differed from the expert raters, participants were more likely to rate themselves higher compared to the expert rater. These are not unsurprising findings since the tool was designed to capture elements that may be overlooked by even experienced research team members. However, this has implications for the utility of the tool for self-assessment and may only be potentially appropriate for secondhand assessment. Overall parental experiences with the BRIEF intervention and assessment tool were positive, and it has been previously noted that poorer scores on POs were associated with declining participation, providing evidence for construct validity of these POs [8]. The ICC was significant for POs, demonstrating internal consistency among POs. Five of the POs had moderate or higher inter-rater reliability from the expert raters. The BRIEF assessment tool was crafted with significant literature, expert, and parental feedback, and feedback from participating research team members suggests the elements with fair or less agreement are still integral to include, pending further collection of validity evidence and a larger sample size [8,9].

The BRIEF educational intervention, including the BRIEF assessment tool, was previously found to be helpful by the research team [9]. The BRIEF assessment tool allows for a continuum of feedback to be provided without a pass/fail designation. The use of the tool within different institutional and clinical trial settings is anticipated to establish validity across a wide range of settings. As the tool is implemented into practice, it may be revised to improve ability for self-assessment. Therefore, caution should be used for relying on self-ratings in its current state given the low correlation between self and expert ratings across domains.

Limitations

This was a pilot study, and a limitation of our study is its small size (n = 5) and homogeneity of our group consisting of all physicians who identified as white. Ideally, we would have had a larger sample size with a more diverse participant population in regard to both role and demographics. Another limitation of this tool is the poor correlation between the participant self-assessment and participant self-reported skill level with the expert raters’ assessment scores. This likely indicates limited utility of the tool in its current state for self-assessment; however, it highlights that the tool was intended to identify areas for improvement. Five of our items had fair or less interrater reliability; with a larger sample size, there may be an increase in the level of agreement of those other elements. With the instruction to expert raters that they may select half-levels, this meant up to nine different assessment scores could be provided per PO, and this large selection choice may affect our interrater reliability. There is always a risk of conscious or unconscious bias concerning the subjectivity of raters, particularly as raters were not blinded to participants’ identity due to the reliance on voice recordings; future ratings could use transcripts rather than audio-recordings to reduce bias. As this is the first proposed validity study examining evidence for a tool to be used in neonatal clinical research team education for enrolling and consenting families, there was no “gold standard” to compare our participant results to; thus, surrogate measures including parental perceptions were used.

## Conclusions

In this pilot study, our tool demonstrated strong preliminary evidence for validity when used as an observer-rated measure by expert raters. Comparatively, there was weaker evidence for use for participant self-assessment. However, these findings are based on a limited pilot study and require further investigation. Additional validation is necessary before the tool can be used for high-stakes purposes, such as pass/fail determinations or outcomes-based assessment.

## Appendices

**Table 5:** BRIEF Assessment Tool BRIEF Assessment Tool: Building Relationships, Informed consent, Effective communication Framework Assessment Tool Each performance objective domain (PO.1-PO.10) is scored on a five-point Likert scale from 1 (Novice) to 5 (Expert), with corresponding observable learner behaviors and illustrative examples at each level. This table has been created by the authors of the present study based on Weiss ​​​​​​et al. [7], which is an open-access article licensed under a Creative Commons Attribution 4.0 International License (http://creativecommons.org/licenses/by/4.0/)

PO.1	Partnership with clinical team: research team member demonstrates to the family that the study team is partnered with the patient’s clinical team to show the link between research and clinical care				
Level	1 (Novice)	2	3	4	5 (Expert)
Behavior	Does not have opportunity to connect with clinical team or discuss this with family	Shares that clinical team is aware of research team’s approaching the family	Connects with clinical team and shares that they support the research team in approaching the family	Illustrates connection between the clinical and research teams by naming who they connected with before approach	Illuminates a strong connection between the clinical and research teams by naming provider and their belief in patient eligibility
Examples	Unable to connect with providers about the research study before approaching family	“I connected with your doctor about your potential eligibility for our study.”	“Your doctor said it was alright if I come talk with you about the study.”	“Dr. Smith said it was OK if I come talk with you about the study.”	“Early today I talked with Dr. Smith, who agreed that your baby might be a good candidate for this study”
PO.2	Partnership with Bedside Nursing: Research team member builds on relationship between family and bedside nursing to support introductory relationship-building between family and the research team.				
Level	1 (Novice)	2	3	4	5 (Expert)
Behavior	Does not have opportunity to connect with nursing or discuss this with family	Describes nursing support for research team presence at bedside	Connects with nursing before approach and tells family they have done so	Illustrates partnership with the bedside nurse by sharing that they support the research team approaching the family	Builds on the connection between the family and nursing, by naming the nurse and sharing with parents information gained from connecting with them
Examples	Unable to connect with bedside nursing about the research study before approaching family	“Nursing knows that research teams like ours often approach families in the NICU.”	“I let your nurse know we’d be chatting about a research study.”	“I let your nurse know we’d be chatting about a research study, and they said this would be a good time.”	“Your nurse Jenny said that now might be an ok time to talk about a potential research study. She mentioned that you guys prefer to keep it quiet in Johnny’s room so I will talk quietly.”
PO.3	Family Names: Research team member initiates relationship with infant and caregivers by learning how they would like to be addressed and addressing them accordingly.				
Level	1 (Novice)	2	3	4	5 (Expert)
Behavior	Proceeds without asking names/roles or omits names during consent conversation	Primarily uses generic titles or roles (ex: “mom”) in conversation	Variably recalls and uses individuals’ preferred terms	Requests and consistently uses individuals’ preferred terms, pronunciations, and pronouns throughout	Plans ahead by obtaining names, then confirms and uses preferred terms, pronunciations, and pronouns
Examples	“Hi, I’m here to talk to you about your baby being in a clinical trial.”	“Are you mom? Great! It’s nice to meet you. Since you are a mom of a baby in the NICU we would like to talk with you about your baby being in a clinical trial.”	“What is your name? It is so nice to meet you, Karen. Since you are a mom of a baby in the NICU, we would like to talk with you about your baby being in a clinical trial.”	“What is your name? It is so nice to meet you, Karen. And who is this? (Referring to baby). As Johnny’s mom, we would like to talk with you about Johnny possibly being in a clinical trial on babies in the NICU.”	“Hello, you must be Johnny’s mom? Is it alright if I call you Karen, or would you prefer Ms. Thompson, or something else? Great! So, Karen, I am here today to talk with you about Johnny possibly being in a clinical trial.”
PO.4	Options for Discussing Research: Research team member provides families choices to make the discussion of research most productive and comfortable for them.				
Level	1 (Novice)	2	3	4	5 (Expert)
Behavior	Confirms with family it is an appropriate time to discuss research	Offers specific options of other times and/or locations to discuss research	Acknowledges that the family may not feel ready to discuss research and offers option to talk later	Empathizes how it may be difficult to discuss research in the NICU and partners with the family to make the experience more comfortable	Individualizes discussion of options by anticipating family’s needs, seeking preferences, and following through to meet preferences
Examples	“Is now a good time to hear about a research study?”	“If you want, I can reserve a quiet room for us to chat, and/or I can come back tomorrow if now isn’t a good time”.	“I know you’re going through a lot, so how do you feel about chatting with me about research? If now isn’t the best time, that’s okay, we can also chat tomorrow.”	“It can be hard to talk about research while in the NICU. If now isn’t the right time or place, I could come back later and/or we could find another space to chat. What feels right to you?”	“I’d like to figure out how to make you feel most comfortable in our conversation. From what you’ve told me about your day so far, maybe it would be good for me to give you some space and come back later?”
PO.5	Empathy with the NICU family experience: research team member acknowledges that the NICU is a challenging place for families and supports relationship-building through empathizing with family experiences				
Level	1 (Novice)	2	3	4	5 (Expert)
Behavior	Opportunity to connect with family regarding their NICU experience does not arise or is not recognized	Responds to specific challenges shared by the family	States recognition of the challenges in the NICU	Invites the family to share their NICU experience with open-ended questions and responds with empathetic statements	Anticipates and explores family NICU experience, recognizes and responds to subtle emotional cues, and provides empathic support
Examples	Doesn’t recognize an opportunity to connect emotionally with family	Responds to family sharing that the patient has had a recent infection scare with:* “That sounds so hard.”*	“I know things can be very challenging for babies and families in the NICU.”	“How are you all doing today?... It sounds like it was a long night, I can’t imagine how stressful that was for your family.”	“I have heard from families about how hard their time in the NICU is. How are you all doing? … Driving hours every day to visit your baby sounds exhausting.”
PO.6	Family needs: research team member supports relationship building by identifying, exploring, acknowledging, and addressing family’s needs				
Level	1 (Novice)	2	3	4	5 (Expert)
Behavior	Opportunity to discuss family needs does not arise or is not recognized	Describes existence of resources for families to get needs or questions addressed	Elicits and acknowledges needs; offers to take action to support getting needs addressed	Explores any unmet needs including responding to parental verbal and non-verbal cues; and acts within role to address as appropriate	Anticipates and identifies needs by preparing to meet the family and directly asking parents their needs; ensures the needs get addressed by the team as a whole
Examples	No family needs arise or are identified	“The hospital has lots of resources available to help with financial concerns.”	“It sounds like you have questions about next steps for your baby. I can let your doctor know you’d like to talk about that.”	“Can I get you anything before we talk more about the study? … Let me grab you some water, and I will touch base with your nurse to make sure she knows you would like to talk about the new medication schedule.”	“Would you be interested in a resource flyer that I have tha toutlines some of the most used and common family resources, including transportation services? Also, would it be okay if I let our social worker Sam know you’d like a visit to discuss options?”
PO.7	Research team’s investment in trial: research team member effectively communicates why this research team is invested in this clinical trial				
Level	1 (Novice)	2	3	4	5 (Expert)
Behavior	Describes the overall goals of this trial	Discusses importance of this trial for the NICU community	Illustrates specific examples of why this trial is valuable to the NICU community	Shares personal reasons why research team members are motivated to do this trial	Formulates a compelling message on how this trial fits within the larger goals of the research team
Examples	“This research aims to determine if iron may impact babies’ brain development.”	“Iron is important for brain development and this research study should teach us how to help more babies in the NICU.”	“The team doing this study are experts in iron metabolism and we are all really hopeful that this proves to be helpful to babies in the NICU.”	“The research team was inspired to address iron as it’s so important to the healthy development of babies, like Johnny. Our team is hopeful that this trial will give us another tool to help babies in the NICU thrive.”	“This study is led by a leader in nutritional support for babies like Johnny who are struggling with the challenges of prematurity. The team and I are hopeful that this project will contribute to our mission of helping babies grow in the NICU.”
PO.8	Benefit for future infants: research team member shares the rationale for the study including jargon-free explanations of why this issue is important to infants and families. Use this explanation to connect the family’s hopes for their own infant with their hopes for other infants in their situation				
Level	1 (Novice)	2	3	4	5 (Expert)
Behavior	States that research can help future infants	States potential benefits of this trial for future infants	Illustrates the connection between research goals and family goals of helping future infants	Proposes a shared goal between family and research team to help infants in the future	Formulates a compelling message of this study’s potential impact on NICU research and connects the message to the individual infant and family
Examples	“Research can help future NICU babies.”	“This trial will teach us about how to manage iron supplementation for future babies in the NICU.”	“We are hopeful to learn from families like yours how to better manage iron supplementation for babies in the NICU.”	“We are hopeful that this trial will help Johnny and other babies like Johnny in the future have the best development they can.”	“We are hopeful that this trial will enable NICU babies like Johnny to thrive. For some families, participation is a way of giving hope to other families in the same situation."
PO.9	Options for participation: research team member demonstrates that the decision whether to participate is the family’s choice and that families make different choices				
Level	1 (Novice)	2	3	4	5 (Expert)
Behavior	Lists the potential risks and benefits to participating in the study and offers an option to participate	Describes potential risks and benefits to participation and ensures family understands it’s an individual choice	Explains that different families might think about the potential risks and benefits differently	Shares potential risks and benefits and compares how different families might apply that information to their decision	Individualizes discussion by eliciting family’s values and experience to support their decision to participate or not participate
Examples	Lists risks and benefits, then tells family they can choose whether to participate or not	“I just shared a lot of things to think about. As you’re making your decision, you can think about which of these things are most important to your family.”	“Families weigh these risks and benefits differently. Some families see the risks of the study as outweighing the benefits, and others see the benefits as outweighing the risks.”	“For some families, the time required to do the research activities may feel overwhelming. For others, it may be seen as a way to get more information about their baby’s development.”	“Each family makes the best decision for them about joining research studies, and they also have different approaches to making that decision. After hearing about the study, what stands out as important for your family?”
PO.10	Ongoing connection with family: research team member provides the information families need to make it easy and comfortable for them to follow up with the research team				
Level	1 (Novice)	2	3	4	5 (Expert)
Behavior	Points out research team contact information on study documents and states they can use it to contact the team	Encourages family to contact research team with any questions using the contact information provided	Acknowledges that families often have questions after the research discussion and expresses research team’s receptivity	Illustrates an example of questions that families may have and highlights how research team can help answer questions	Identifies potential areas of concern based on family’s expressed values and ensures family knows how research team can help
Examples	“Our study team’s information is listed at the top of the study consent form if you have any questions or concerns, you can contact us.”	“Our information is at the top of the study consent form. Please reach out to us with any questions. That’s what we’re here for.”	“Families often have questions about the research that come up later. Our contact information is here and we encourage you to ask us any questions you have.”	“Sometimes questions come up later. For example, families will wonder about the timeline for the study activities. Our team can answer all of those questions for you.”	“You mentioned you had questions about our access to your baby’s information. If you want more detailed information, I can ask the study doctor to come talk to you now, or if things come up later you can reach us at the number listed here.”
